# *ReadSeeker*: A DNABERT based de-novo read-level gene predictor

**DOI:** 10.1371/journal.pone.0335732

**Published:** 2025-11-13

**Authors:** Ben Wulf, Piotr Wojciech Dabrowski

**Affiliations:** Center for Bio-Medical Image and Information Processing (CBMI), HTW University of Applied Sciences, Berlin, Berlin, Germany; Emory University, UNITED STATES OF AMERICA

## Abstract

ReadSeeker, a newly fine-tuned, DNABERT-based model, differentiates NGS short reads into protein-coding (CDS) and non-protein-coding (non-CDS) categories without requiring known reference sequences. For model training, extensive datasets encompassing viral, bacterial, and mammalian sequences where used. Training involved generating approximately 3 million synthetic reads from annotated genomic elements.

Performance evaluation on real-world datasets, including human, viral, and bacterial samples, revealed ReadSeeker’s high accuracy, exceeding 94%, with ROC-AUC scores above 98% in most cases.

## Introduction

The advent of Next-Generation Sequencing (NGS) technologies has catalyzed a transformative shift in the landscape of genomics, offering unprecedented insights into the complexity of biological systems.

However, the amount of data generated using NGS is bringing classical rule-based analysis methods to their limits. In those areas where weak signals and deeply hidden patterns must be recognized in order to understand the data, novel approaches are needed.

The remarkable success of deep learning models, especially those inspired by the transformer architecture, in various fields such as natural language processing, is opening new avenues. The BERT (Bidirectional Encoder Representations from Transformers) model, in particular, has demonstrated exceptional proficiency in understanding complex patterns in large text corpora [1].

Drawing inspiration from this, we introduce our novel fine-tuned deep learning model (*ReadSeeker*) based on the DNABERT model [2], which is highly optimized on genomic classification tasks.

*ReadSeeker* is able to separate NGS reads of lengths between 151-301 base pairs (bp) into those stemming from protein coding (CDS) and non-protein coding (nonCDS) regions, without explicitly relying on known reference sequences or additional information such as the presence of start or stop codons. *ReadSeeker’s* superior classification performance, compared to existing methods like *FragGeneScan* [3], can greatly enhance its utility in various NGS pipelines.

## Materials and methods

### Data training

*ReadSeeker* is optimized for CDS/nonCDS discrimination of viral, bacterial and mammalian sequences. All viral and bacterial sequences annotated with *“Full”* as genome representation and *“Complete Genome”* at assembly level were obtained from the NCBI Reference Sequence Database (RefSeq [4], retrieved on March 25^th^ 2024). This resulted in 14.519 viral genomes and 38.137 bacterial genomes. Additionally, the latest releases of the *Human* (*GCF_000001405.40_GRCh38.p14*), *Pig* (*GCF_000003025.6_Sscrofa11.1*) and *Greater horseshoe bat* (*GCF_004115265.2_mRhiFer1_v1*) were included in the dataset.

Using custom Python scripts, the reference genomes were segmented into contiguous regions of at least 300 base pairs, classified strictly as either CDS or nonCDS. This classification was based on the “CDS” annotations within the respective GenBank entries. Regions not annotated as CDS were designated as non-coding. Regions with fewer than 300 bp were discarded.

For model training, 2,999,996 random reads were generated from both CDS and nonCDS segments, with an additional 29,996 reads allocated for training evaluation. Each synthetic read was precisely 300 bases in length. Stratified random sampling was employed to ensure that each reference category - viral, bacterial and mammalian (human/pig/bat), - contributed equally to the CDS and nonCDS pools. Reads containing ambiguous nucleotide symbols (e.g., ‘N’, ‘Y’) were excluded and replaced by another randomly selected read from the same category and sequence type. The probability of selecting a specific genomic region for read generation was weighted by its length relative to the total length of all regions within the same reference category and coding status. The training and evaluation datasets were shuffled to prevent potential biases associated with sequence order, thereby minimizing the risk of temporal over-fitting. Detailed distributions of train datasets are presented in Table 1 and supplemental S1 Fig, the scripts used to perform the dataset preparation are available on GitHub [5].

**Table 1:** Overview training dataset.

Reference Category	# CDS reads	# nonCDS reads	# total
viral	500.000	500.000	1.000.000
bacterial	500.000	500.000	1.000.000
mammal/human	166.666	166.666	333.332
mammal/pig	166.666	166.666	333.332
mammal/bat	166.666	166.666	333.332
total	1.499.998	1.499.998	2.999.996

The table displays the breakdown of sampled reads for training the *ReadSeeker* model, categorized by reference (viral, bacterial, mammalian/human, mammalian/pig, mammalian/bat) and coding type (*CDS*, *nonCDS*). It provides detailed counts for each category along with the overall totals, illustrating the representation and balance achieved in the training dataset.

Building upon the dataset preparation described, the fine-tuning of the *ReadSeeker* model leveraged the DNABERT 6-mer model (*k* = 6), which had previously demonstrated superior classification performance on prediction of splice donor and acceptor sites and highest loss during pre-training among the DNABERT suite of models [2]. The fine-tuning process was conducted using the scripts available from the DNABERT resources, with specific parameter adjustments to optimize performance. The learning rate was set at 2e-5, with the model undergoing five training epochs. A warmup percentage of 0.1 was applied to gradually adjust the learning rate, alongside a hidden dropout probability of 0.1 to prevent overfitting. Additionally, a weight decay parameter of 0.01 was included to further regulate the model complexity. The maximal sequence length was capped at 298 tokens.

The fine-tuning process was conducted on a single *NVIDIA A40-24Q* GPU equipped with 24 GB of memory, and it required approximately 72 hours to complete.

### Performance evaluation on real world datasets

To assess the *ReadSeeker* model’s efficiency on real-world data, six comprehensively annotated reference genomes were selected: two bacterial and two viral genomes, mouse and human genome. A total of eleven publicly available datasets were utilized for performance evaluation, comprising three *human* stool samples, two *SARS-CoV-2* samples, one *Epstein-Barr Virus* (EBV), three *Mycobacterium tuberculosis*, one *Escherichia coli* and one *Mus musculus* sample, as detailed in Table 2. The *SARS-CoV-2* samples where chosen to illustrate the classifier behavior on compact genomes with high gene density.

**Table 2:** Overview test dataset.

Reference Category	Reference	Sample	Uniprot Taxon	cds balancing	# Reads	Read length
mammal	human (GRCh38)	ERR10492982	-	balanced	31642	251
mammal	human (GRCh38)	ERR10493241	-	balanced	32084	251
mammal	human (GRCh38)	ERR10509672	-	balanced	24262	251
mammal	Genomic Benchmark (human)	coding/intergenomic	-	balanced	25000	200
mammal	Mus musculus (NC_000067.7)	DRR317657	-	balanced	25926	255
bacterial	M.tuberculosis (NC_000962.3)	SRR21820122	1762	balanced	9718	301
bacterial	M.tuberculosis (NC_000962.3)	SRR21820124	1762	balanced	9884	301
bacterial	M.tuberculosis (NC_000962.3)	SRR21864655	1762	balanced	12576	301
bacterial	E.Coli (NC_000913.3)	SRR22674487	561	balanced	16624	151
viral	EBV (GCF_002402265)	ERR2024408	548681	balanced	776508	300
viral	SARS-CoV-2 (NC_045512.2)	ERR10913059	-	unbalanced	427828	301
viral	SARS-CoV-2 (NC_045512.2)	ERR10913061	-	unbalanced	311974	301

The Uniprot Taxon is the taxon used to download Uniprot protein sequences to treat potential false positive hits. CDS balancing indicates if the sample was subsampled, such that the number of coding and non-coding reads is identical. # Reads shows the total number of reads used for benchemarking. # Reads consists of 50% CDS and 50% nonCDS read for balanced Datasets. Each of the SARS-CoV2 samples had 4 nonCDS reads. Read length is the maximal read length in the dataset.

The mouse dataset was subset to the first 10 million reads, resulting in 25926 filtered and balanced reads in the benchmark.

To mitigate the impact of missing gene annotations in the reference genomes, higher taxonomic groups were manually selected for downloading protein sequences for *Mycobacterium tuberculosis*, *Escherichia coli*, and *EBV*, as noted in (Table 2. Protein sequences corresponding to the UniProt [6] taxonomic identifiers were downloaded and searched against the respective reference genomes using *tblastn* [7]. A *tblastn* hit was treated as possibly coding if the hit had a sequence identity of greater than 90% and and the Protein alignment size was greater than 75% of the protein size. Regions that were annotated as CDS and those considered as possible coding were merged to generate a mask to exclude those regions from the nonCDS evaluation.

For accurate alignment, all samples were mapped to their respective reference genomes using *Bowtie2* [8], as referenced in Table 2. To ensure a high quality of alignments, only reads with a mapping quality of 42 or higher were considered. *Samtools* [9] and *BEDTools* [10] facilitated the identification of reads that were entirely within a CDS region and those completely outside any known CDS or unclear regions. Reads that overlapped partially with CDS regions were excluded from the analysis.

Except for the *SARS-CoV-2* samples, which lacked sufficient non-coding regions to generate nonCDS reads, the datasets were balanced to contain an equal number of coding and non-coding reads. The sampling was carried out randomly from the larger group to maintain this balance.

Additionally to our own datasets, we added the human transcriptome based “Genomic Benchmark - demo_coding_vs_intergenomic_seqs” [11] testdataset to our benchmark comparison.

Furthermore, we benchmarked *FragGeneScan* [3] in version 1.32 released in December 2024 and *Genomic Benchmark - Simple Base Model* [11] in comparison to *ReadSeeker*. *FragGeneScan* was executed utilizing the ‘illumina_5’ model, which assumes an error rate of 0.5% in the reads. The execution was configured with the option ‘-w 0’ to accommodate short reads and used 50 CPU cores.

To obtain the *Genomic Benchmark - Simple Base Model*, we retrained the model on “Genomic Benchmarks - demo_coding_vs_intergenomic_seqs” training sequences according to published scripts and evaluated the model on 50 CPU cores with our benchmark datasets.

*ReadSeeker* was executed on a NVIDIA A40-24Q GPU with 24 GB of memory during our benchmark tests, with exception for the “Genomic Benchmark - demo_coding_vs_intergenomic_seqs” dataset, which was executed on 50 CPU cores.

In order to maximize the reproducibility of the evaluation process, the processing of real-world read data was comprehensively conducted utilizing a custom *Snakemake* [12] pipeline. The final visualization of the classification results was achieved through the utilization of custom Python scripts in a jupyter notebook.

For evaluation purposes, a *ReadSeeker* decision threshold of ≥ 0.5 was used for discrimination of CDS reads and nonCDS reads respectively. According to the *FragGeneScan* manuscript [3], a read is classified as a coding sequence (CDS) if at least 50% of its bases are identified as CDS.

The *ReadSeeker-Model* and scripts to generate training and evaluation data are available on GitHub [5]. The training and blast datasets are available at Zenodo [13].

## Results and discussion

**Fig 1:**
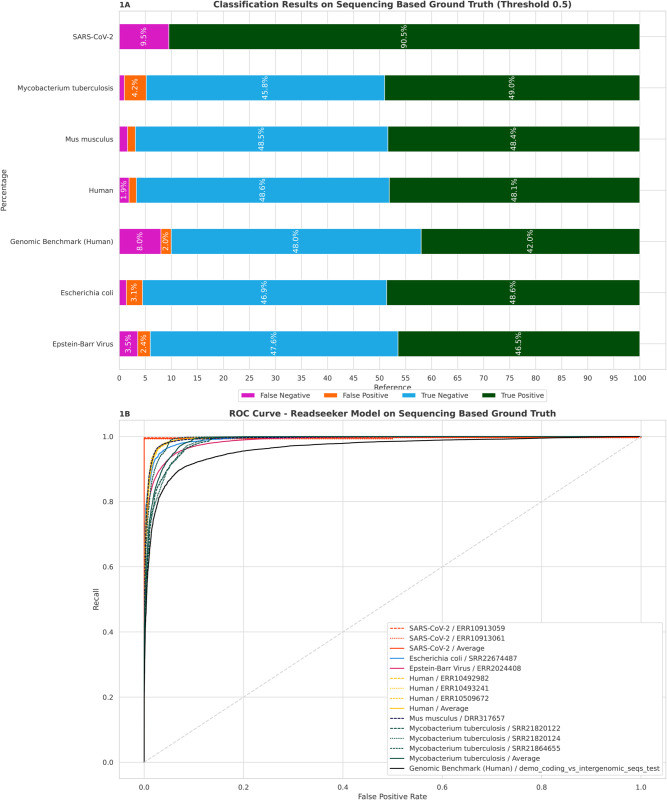
Performance of ReadSeeker on 11 NGS sequenced Datasets and the “Genomic Benchmark coding/intergenomic dataset”. **1A)** *ReadSeeker* classification using a discrimination threshold of ≥ 0.5. The independent result samples show accuracies of up to 94% and comparable proportions of false positives and false negatives. Due to the different genome structure, the *SARS-CoV-2* samples have no displayable negative *nonCDS* results. 1 **B)** ROC-Curves of the *ReadSeeker* classification. The colors summarize the different reference sequences. The average ROC curves generated per reference and for individual samples are represented by solid lines. In the case of multiple samples, the individual samples are shown dashed and faded. The ROC-AUC is at least 96.4% (*Genome Benchmark - Human*). See Table 3.

As previously described, we benchmarked our newly fine-tuned *ReadSeeker*-model, *FragGeneScan* and the *Genomic Benchmark - Simple Base Model* on 6 different sources of genetic material. *ReadSeeker* predicted the correct coding/non-coding classes with an accuracy of at least 90.0% (“Genome Benchmark” dataset) and 96.9% (*Mus musculus*) (Table 3, Fig 1A, S2 Fig). Furthermore, the *ROC-AUC* (Receiver Operating Characteristics, Area Under The Curve) of our *ReadSeeker* model is greater than 96,40% (“Genome Benchmark” dataset) for all tested sample groups and reaches up to 99.58% (*SARS-CoV-2*) (Table 3, Fig 1B). *ReadSeeker* achieves F1-Scores between 89.40% (“Genome Benchmark” dataset) and 96.91% (*Mus musculus*). The model specific Matthews Correlation Coefficients (MCC) range from 0.8066 (“Genome Benchmark” dataset) to 0.938 (*Mus musculus*).

**Table 3:** *ReadSeeker*-model, *FragGeneScan* and Genomic Benchmark - *Base Model* benchmark metrics.

Reference	Tool	Accuracy	ROC-AUC	F1	MCC
*EBV*	*ReadSeeker*	**0.9406**	**0.9874**	**0.9399**	**0.8814**
	*FragGeneScan*	0.7552	0.8701	0.7942	0.5516
	*Simple Base Model*	0.7170	0.8753	0.7734	0.5005
*SARS-CoV-2*	*ReadSeeker*	0.9049	**0.9958**	0.9501	0.0101
	*FragGeneScan*	**0.9922**	0.9904	**0.9961**	-0.0003
	*Simple Base Model*	0.4734	0.7327	0.6426	0.0031
*E. coli*	*ReadSeeker*	**0.9555**	**0.9925**	**0.9562**	**0.9115**
	*FragGeneScan*	0.7012	0.8093	0.7696	0.4999
	*Simple Base Model*	0.6941	0.9017	0.7618	0.4718
*M. tuberculosis*	*ReadSeeker*	**0.9482**	**0.9884**	**0.9499**	**0.8984**
	*FragGeneScan*	0.7455	0.8398	0.7946	0.5590
	*Simple Base Model*	0.5013	0.9017	0.6673	0.0366
*Human*	*ReadSeeker*	**0.9672**	**0.9943**	**0.9671**	**0.9345**
	*FragGeneScan*	0.7051	0.8185	0.7551	0.4493
	*Simple Base Model*	0.8176	0.9022	0.8050	0.6406
*Mus musculus*	*ReadSeeker*	**0.9692**	**0.9936**	**0.9692**	**0.9384**
	*FragGeneScan*	0.7374	0.8658	0.7898	0.5476
	*Simple Base Model*	0.7419	0.8410	0.7031	0.5012
*GB (Human)*	*ReadSeeker*	**0.9004**	**0.9640**	**0.8940**	**0.8066**
	*FragGeneScan*	0.6473	0.7376	0.7134	0.3320
	*Simple Base Model*	0.8899	0.9584	0.8923	0.7806
*Overall*	*ReadSeeker*	**0.9266**	**0.9814**	**0.9471**	**0.8327**
	*FragGeneScan*	0.8529	0.8808	0.9046	0.6189
	*Simple Base Model*	0.6151	0.6093	0.7097	0.1541

For each reference group the table shows *ReadSeeker’s*, *FragGeneScan’s* and the *Genome Benchmark - Simple Base Model’s* Accuracy, Receiver operating characteristic - Area Under Curve (ROC-AUC), F1-Score and Matthews Correlation Coefficient (MCC) based on classification results of the short reads samples. Best Values are highlighted in bold. MCC values for the SARS-Cov-2 Samples are highly affected by the by the unbalanceable genome structure of the SARS-CoV-2 genome leading to a low number of 8 non-coding reads. Even perfect classifiers receive a score of 0 with a completely unbalanced data set. Beside the unbalanced *SARS-CoV-2* dataset, *Readseeker* outperforms *FragGeneScan* and the *Genome Benchmark - Simple Base Model* in all classification performance measurements. The *Genome Benchmark - Simple Base Model* showed lower accuracy on all datasets, which are not related to the “Genome Benchmark” dataset, indicating a potential overfit.

The *SARS-CoV-2* dataset exhibited an unusually low MCC score of 0.01, suggesting that the classifier’s decisions are close to random guessing. This outcome is attributed to the highly imbalanced nature of the *SARS-CoV-2* genome, which resulted in only 8 non-CDS reads of 739802 reads in the dataset (S2 Fig). Such imbalance in the dataset activates the MCC’s characteristic of interpreting datasets with predominantly one-sided distributions,in this case mostly coding, as the equivalent of random decisions [14].

*ReadSeeker* missclassifed 8% of the “Genomic Benchmark - demo_coding_vs_intergenomic_seqs” dataset as false-negative (Fig 1). In comparison that is more than four times the amount of false-negative reads in our human dataset. This difference is probably caused by the inclusion of intronic sequences as coding sequences in the “Genomic Benchmark - demo_coding_vs_intergenomic_seqs” dataset, which are treated as nonCDS in our training and evaluation data.

On our balanced benchmark datasets *ReadSeeker* does not show significant differences in generating more false-positive respectively, false negative reads (Fig 1A).

Compared to *ReadSeeker*, *FragGeneScan* demonstrated slightly higher true-positives with its chosen discrimination thresholds (Table 3, S3 Fig). However, *FragGeneScan* classifed approximatly 50% of the nonCDS reads as CDS, resulting in reduced overall performance metrics, including accuracy, ROC-AUC, F1, and MCC scores. An exception was observed in the unbalanced SARS-CoV-2 sample, where *FragGeneScan*, owing to its high sensitivity, achieved superior accuracy (99.2% versus 90.4%) and F1 score (0.99 versus 0.95) (Table 3) compared to *ReadSeeker*.

The *Genomic Benchmark - Simple Base Model* showed the lowest accuracies (Table 3) in comparison to *ReadSeeker* and *FragGeneScan*, while being around two times slower (Sect 0.2) in comparison to *FragGeneScan* (Sect 0.2). Furthermore, the *Genomic Benchmark - Simple Base Model* showed better classification performance metrics on the “Genomic Benchmark - demo_coding_vs_intergenomic_seqs” dataset compared to the other samples, especially our human dataset. This indicates that the model overfit the the training dataset and learned some dataset specific markers. Furthermore, the model is not able to properly classify bacterial sequences (Table 3, S4 Fig). On the *M. tuberculosis* dataset the *Simple Base Model* has an accuracy of 0.5 and an MCC of 0.03 meaning the classification result is random.

In terms of computational efficiency, *FragGeneScan* demonstrates a superior performance, operating approximately 25 times (Sect 0.2) faster than *ReadSeeker*. The results showed that *ReadSeeker* running on a GPU required between 4.91 seconds and 7.01 seconds per 1,000 reads, whereas *FragGeneScan* on CPU only required between 0.20 seconds and 0.27 seconds per 1,000 reads (Sect 0.2). This substantial difference highlights the computational efficiency advantage of the HMM based *FragGeneScan*.

In future projects, the classification speed disadvantage of *ReadSeeker* could be mitigated through model optimization techniques such as pruning, distillation, and quantization. By embracing slight trade-offs in prediction accuracy, these techniques have the potential to significantly reduce the model size and consequently increase inference speed.

The *ReadSeeker* system, alongside its underlying DNABERT model, was initially trained on the human reference genome. This raises a potential concern regarding information leakage when applied to the human test dataset. However, the observation that *ReadSeeker* demonstrated superior classification performance (Accuracy, F1 and MCC) on the mouse dataset suggests that any information leakage into the human test dataset, if present, has a negligible impact.

Due to data availability, the evaluation of *ReadSeeker* was performed on datasets with read lengths in the range of 151 bp to 301 bp (Table 1). *ReadSeeker’s* classification performance on the (*E.coli*) dataset with 151 bp reads does not show a significant difference in the benchmark metrics (Table 3) compared to the datasets with read lengths between 251 bp and 301 bp, indicating a robust classification behavior for read lengths between (151 and 301BP).

The strong performance of the model across different species and various read lengths indicates its robustness and reliability in diverse genomic contexts.

## Conclusion

The results demonstrate that our *ReadSeeker* model is effective in annotating short genomic regions of 300 bp as belonging to CDS or non-CDS genome regions with an accuracy exceeding 94%, except for the unbalanced *SARS-CoV-2* samples and the “Genomic Benchmark - demo_coding_vs_intergenomic_seqs” dataset, created by other interpretation of coding sequences, as discussed previously.

Furthermore, *ReadSeeker* showed superior classification performances in compared to the existing *Genomic Benchmark - Simple Base Model* and *FragGeneScan*. The later showed a lack in specificity (S3 Fig). Unlike existing methods, such as specific Hidden Markov Models (HMM) like *FragGeneScan*, our model does not depend on tracking open reading frames (ORFs), promoter regions from large assemblies, or the direct assignment using closely related reference sequences, as seen with RATT [15]. In contrast to context sensitive Hidden-Markov-Models [16] capable of including a few contextual bases, *ReadSeeker* can use the whole read as context due to its transformer based structure. Additionally, the *ReadSeeker* model exhibits robust performance across diverse organism groups, including viruses, bacteria, and mammals. We could also show a high performance of *ReadSeeker* on organisms with highly unbalanced genomes like the *SARS-CoV-2* Samples.

Although the nature of neural networks renders our *ReadSeeker* model is approximately 25 times slower compared to *FragGeneScan* (Sect 0.2), it serves as a valuable complementary method. This is particularly evident in scenarios where *FragGeneScan’s* accuracy is insufficient and reference-based annotations are inadequate due to high mismatches between target and reference sequences or where reference sequences are incomplete.

The model’s independence from organism-specific constraints allows it to be applied broadly across different fields of genomics, including environmental DNA studies and metagenomics.

Lastly, the integration of the *ReadSeeker* model with existing genomic analysis pipelines can provide a more holistic understanding of genomic data, aiding in the discovery of new genes, regulatory elements, and other functional genomic regions. Future work will focus on optimizing the model’s inference speed and exploring its applicability to even shorter reads than 151BP and a potential change to the Byte-Pair-Encoding based *DNABERT 2* [17].

## Supporting information

S1 Fig Krona plot of training dataset. The Krona plot illustrates the taxonomic distribution of the *ReadSeeker* training dataset, providing a comprehensive overview of the relative abundance of different taxa present in the dataset. The hierarchical structure of taxonomic classifications is depicted, allowing for an intuitive understanding of the taxonomic composition of the dataset The graphic illustrates the even source data distribution on viral (red), bacterial(cyan) and mammalian (green) genomes.
(TIFF)

S2 Fig *Readseeker* Confusion matrices. The confusion matrices illustrate the performance of the *Readseeker* classifier on datasets derived from 12 different samples and evaluated against seven test reference genomes. Here, the label ‘0’ corresponds to non-coding (nonCDS) read sequences, while the label ‘1’ indicates coding sequence (CDS) reads. The results demonstrate that *Readseeker* effectively distinguishes between nonCDS and CDS reads, highlighting its strong sensitivity and specificity. Notably, the classification behavior for the *Sars-CoV-2* dataset deviates from the other reference genomes. This anomaly arises from the dataset’s inherent imbalance, characterized by only 8 nonCDS reads.
(EPS)

S3 Fig *FragGeneScan* Confusion matrices. The confusion matrices display the classification performance of *FragGeneScan* applied to datasets from 12 samples and evaluated against seven test reference genomes. In this context, the label ‘0’ denotes non-coding (nonCDS) read sequences, while the label ‘1’ represents coding sequence (CDS) reads. *FragGeneScan* exhibited a high sensitivity, evidenced by its accurate classification of the majority of CDS reads. However, its specificity was compromised, with nearly 50% of nonCDS reads being misclassified as CDS. This reflects a challenge in distinguishing nonCDS from CDS reads within this dataset. Additionally, the *Sars-CoV-2* dataset demonstrated outlier behavior, attributed to its unbalanced nature, characterized by merely 8 nonCDS reads.
(EPS)

S4 Fig *Genomic Benchmark - Simple Model Confusion* matrices. The confusion matrices display the classification performance of *Simple Base Model* applied to datasets from 12 samples and evaluated against seven test reference genomes. In this context, the label ‘0’ denotes non-coding (nonCDS) read sequences, while the label ‘1’ represents coding sequence (CDS) reads. The*Genomic Benchmark - Simple Model* classified nearly all negative sequences of the M. tuberculosis as coding leading to a random always CDS decision for this dataset. On the *Epstein-Barr Virus* and the *E. coli* samples, the *Genomic Benchmark - Simple Model* misclassified 26.5% to 29.5% false-positives. Additionally, the *Sars-CoV-2* dataset demonstrated outlier behavior, attributed to its unbalanced nature, characterized by merely 8 nonCDS reads.
(EPS)

S1 Table Real process time comparison of *ReadSeeker* and *FragGeneScan.* (PDF)

S2 Table Comparison of the Real Processing Time per 1000 Reads of *ReadSeeker* and *FragGeneScan.* (PDF)
