# Supplementary figures and images for: ReadSeeker: A DNABERT based de-novo read-level gene predictor
Source: PLoS One. 2025 Nov 13;20(11):e0335732. doi: 10.1371/journal.pone.0335732 (PMC12614542; doi:10.1371/journal.pone.0335732)

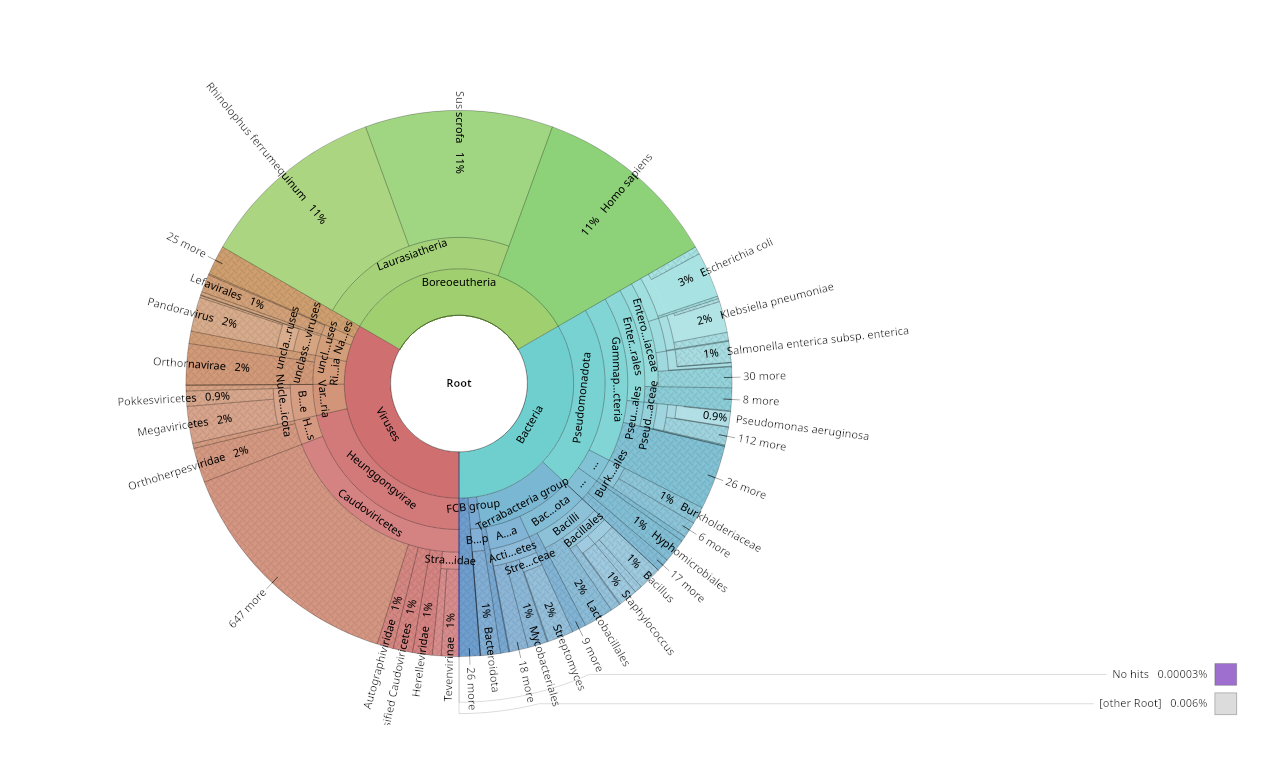

Supplement: S1 Fig — The Krona plot illustrates the taxonomic distribution of the ReadSeeker training dataset, providing a comprehensive overview of the relative abundance of different taxa present in the dataset. The hierarchical structure of taxonomic classifications is depicted, allowing for an intuitive understanding of the taxonomic composition of the dataset The graphic illustrates the even source data distribution on viral (red), bacterial(cyan) and mammalian (green) genomes. (TIFF) [file pone.0335732.s001.tiff]
