# Supplementary material for: ReadSeeker: A DNABERT based de-novo read-level gene predictor
Source: PLoS One. 2025 Nov 13;20(11):e0335732. doi: 10.1371/journal.pone.0335732 (PMC12614542; doi:10.1371/journal.pone.0335732)
Supplement: S1 Table — (PDF) [file pone.0335732.s005.pdf]

S1 Table

Reference	Sample	FragGeneScan	GB Simple Base Model	ReadSeeker	Speed Factor (FGS)	Speed Factor (SBM)
E. coli	*SRR22674487*	3.8s	9.7s	96.9s	25.6x	10.0x
EBV	*ERR2024408*	148.8s	218.5s	3461.2s	23.3x	15.8x
GB (human)	*coding/intergenomic*	5.4s	12.3s	2228.8s	413.5x	180.9x
Human	*ERR10492982*	6.7s	13.7s	171.5s	25.5x	12.5x
	ERR10493241	6.8s	14.1s	173.3s	25.4x	12.3x
	ERR10509672	5.4s	12.0s	135.1s	25.2x	11.2x
M. tuberculosis	*SRR21820122*	2.6s	8.9s	67.0s	26.1x	7.5x
	SRR21820124	2.7s	8.1s	69.2s	25.8x	8.5x
	SRR21864655	3.1s	8.9s	79.8s	25.8x	8.9x
Mouse	*DRR317657*	5.9s	12.1s	143.9s	24.5x	11.9x
SARS-CoV-2	*ERR10913059*	86.0s	156.7s	2105.0s	24.5x	13.4x
	ERR10913061	62.2s	115.0s	1531.2s	24.6x	13.3x

The values in the “FragGeneScan”, “ReadSeeker” and “Genomic Benchmark Simple Base Model” column represents the real process time in seconds. “Speed Factor(FGS)” and “Speed Factor(SBM)” are the relative runtimes of *FragGeneScan* and “Genomic Benchmark Simple Base Model” compared to *ReadSeeker*. On average *ReadSeeker* is around 25 times slower on a *NVIDIA A40-24Q* with 24GB memory compared to *FragGeneScan* and 8 to 15 times slower on *Intel(R) Xeon(R) Gold 6130* with 50 Cores. *ReadSeeker's* evaluation on the *Genomic Benchmark dataset* (“GB (human)”) was performed on the same *Intel(R) Xeon(R) Gold 6130* with 50 Cores, causing outling high speedfactors.
