# Supplementary material for: ReadSeeker: A DNABERT based de-novo read-level gene predictor
Source: PLoS One. 2025 Nov 13;20(11):e0335732. doi: 10.1371/journal.pone.0335732 (PMC12614542; doi:10.1371/journal.pone.0335732)
Supplement: S2 Table — (PDF) [file pone.0335732.s006.pdf]

S2 Table

Reference	Sample	# Reads	FragGeneScan	GB Simple Base Model	ReadSeeker
E. coli	SRR22674487	16624	0.23s	0.58s	5.83s
EBV	ERR2024408	776508	0.19s	0.28s	4.46s
Genomic Benchmark (human)	coding/intergenomic	25000	0.22s	0.49s	89.15s
Human	ERR10492982	31642	0.21s	0.43s	5.42s
	ERR10493241	32084	0.21s	0.44s	5.40s
	ERR10509672	24262	0.22s	0.50s	5.57s
M. tuberculosis	SRR21820122	9718	0.26s	0.92s	6.90s
	SRR21820124	9884	0.27s	0.82s	7.01s
	SRR21864655	12576	0.25s	0.71s	6.35s
Mouse	DRR317657	25926	0.23s	0.47s	5.55s
SARS-CoV-2	ERR10913059	427828	0.20s	0.37s	4.92s
	ERR10913061	311974	0.20s	0.37s	4.91s

The table presents a comparison of the real processing time required by *Readseeker*, *FragGeneScan* and the "Genome Benchmark - Simple Base Model" . The column labeled '# Reads' indicates the number of reads for each sample. The columns for *FragGeneScan*, *GB Simple Base Model* and *Readseeker* display the real processing time measured per 1,000 reads. For the "Genomic Benchmark (human)" dataset, *ReadSeeker* was evaluated on CPU instead of GPU causing an increased relative runtime.
